# High-Resolution and Large-Dynamic Range Fiber-Optic Sensors Based on Dual-Mode Direct Spectrum Interrogation Method

**DOI:** 10.3390/s24123996

**Published:** 2024-06-20

**Authors:** Min Zhou, Zhe Zhang, Qingyue Cui, Qingdian Lin, Jun Yu, Xiaoyang Guo, Cangtao Zhou, Shuangchen Ruan

**Affiliations:** 1 Shenzhen Key Laboratory of Ultra-Intense Laser and Advanced Material Technology, Center for Intense Laser Application Technology, and College of Engineering Physics, Shenzhen Technology University, Shenzhen 518118, China; zhoumin@sztu.edu.cn (M.Z.); 2210412016@email.szu.edu.cn (Q.C.); linqingdian@sztu.edu.cn (Q.L.); yujun@sztu.edu.cn (J.Y.); zcangtao@sztu.edu.cn (C.Z.); scruan@sztu.edu.cn (S.R.); 2 Sino-German College of Intelligent Manufacturing, Shenzhen Technology University, Shenzhen 518118, China

**Keywords:** Fabry-Perot interferometer, chirped fiber Bragg gratings, dual-mode spectrum interrogation method

## Abstract

Conventional optical fiber temperature/strain sensors often have to make compromises between the resolution and the dynamic range. Here we present a new method that meets the measurement requirements for both high resolution and large dynamic range. A high-quality optical fiber Fabry-Perot Interferometer (FPI) constructed using a pair of chirped fiber Bragg gratings is employed as the sensor and a dual-mode direct spectrum interrogation method is proposed to identify the small drift of external temperature or strain. As a proof-of-concept illustration, a temperature resolution of 0.2 °C within 30–130 °C is demonstrated. For strain sensing, the resolution can be 10 µε within 0–1000 µε. The measurement resolution can be improved further by routinely increasing the reflectivity of the CFBG and the cavity length and the sensor can also be mass-produced. This new sensing schema not only resolves the conflict between the resolution and the dynamic range of fiber-optic temperature/strain sensors but can also be extended to other sensors and measurands.

## 1. Introduction

Temperature and strain are two of the basic physical parameters that need to be precisely determined in a lot of application scenarios. Fiber FPIs have been extensively studied for sensing applications due to their superiorities such as high sensitivity, small size and weight, excellent suppleness, immunity to electromagnetic interference, and embedded measuring abilities [1,2,3,4,5]. For temperature or strain sensing, resolving the wavelength of the resonant peak or dip of FPIs is commonly adopted, where the resolution is limited by the bandwidth Δλ of the resonant peak/dip within the spectra [6]. Early works have demonstrated that the resolution and detection limit of a fiber-optic sensor are closely related to the Q-factor by Q=λ/Δλ [6] (Δλ is the bandwidth of the resonant peak/dip). Thus, narrowing the bandwidth (Δλ) of the resonant peak/dip within the spectrum can greatly improve the resolution, followed by the detection limits. Recently, we narrowed the spectrum bandwidth Δλ (i.e., improved the *Q* factor) of an opened air-cavity FPI gas pressure sensor by lengthening the cavity, where a pressure detection limit of 23 Pa is demonstrated [7]. However, the narrowing of the spectrum bandwidth Δλ also minifies the free spectrum range (FSR) of the interference spectrum, resulting in reduced dynamic measurement range. This indicates that the detection resolution and dynamic range of conventional FPI sensors are mutually restrictive.

In-fiber Bragg gratings have been extensively employed in sensing applications [8,9,10]. The dynamic range for temperature and strain can be larger than 1000 °C [11] and 1000 µε [12], respectively. However, the resolution is limited by the large bandwidth (0.1–1 nm) of the FBG. Early works have constructed FPI sensors using two similar FBGs inscribed nearby [13,14,15], resulting in a narrower bandwidth of the tagged peak/dip within the spectrum while the FSR of the spectrum is reduced accordingly, restricting the dynamic range of the sensor. As such, new spectral interrogation methods are requisite for simultaneous high-resolution and large dynamic range sensing applications. In 2015, Liu et al. developed an average wavelength tracking method for a micro-fabricated fiber FPI sensor [16], where a temperature resolution of 6 × 10^−4^ °C is demonstrated. However, the fabrication in micron dimension and the complicated data processing constrain its practical applications. An FPI temperature sensor formed by a pair of FBGs was proposed by Li et al. in 2021 [17]. They replaced the conventional peak/dip wavelength resolving demodulation approach with laser frequency dither locking schema, by which a resolution of 7 × 10^−4^ °C within a dynamic range of ~46 °C is achieved. However, the method needs to convert the direct wavelength shift into the feedback voltage, where expensive source (DFB laser) and complex circuits, feedback, and control parts (lock-in amplifier, proportional-integral-derivative controller, etc.) are indispensable.

In this work, we propose and demonstrate a dual-mode direct spectrum interrogation approach for simultaneously high-resolution and large-dynamic range temperature or strain sensing. The sensor is constructed using a pair of Chirped Fiber Bragg Gratings (CFBGs) inscribed using an ultraviolet (UV) laser phase mask scanning setup. The prepared sensor yields a high *Q* factor of ~0.67 × 10^5^. As a proof-of-concept, a temperature resolution of 0.2 °C within 30–130 °C is experimentally demonstrated. For strain sensing, the resolution can be 10 µε within 0–1000 µε. The proposed sensing schema not only resolves the conflict between the resolution and the dynamic range but simplifies the sensing system, which may find applications in in vivo temperature measurement of living organisms, ultrasonic hydrophones for medical sensing, and even seismic sensors for geophysical surveys.

## 2. Principle and Sensor Design

**Figure 1:**
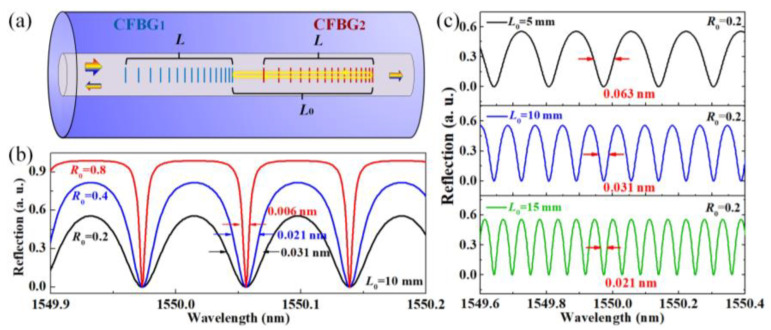
(**a**) Schematic of the proposed FPI sensor, the iridescent arrows represent the incident and reflected light, respectively; (**b**) The simulated reflection spectra of three FPI sensors with CFBG reflection *R*_0_ of 0.2, 0.4, and 0.8 (the cavity length *L*_0_ is assumed to be 10 mm); (**c**) The simulated reflection spectra of three FPI sensors with cavity length *L*_0_ of 5 mm, 10 mm, and 15 mm (the CFBG reflection *R*_0_ is assumed to be 0.2 mm).

The proposed FPI sensor that was constructed using a pair of CFBGs is sketched in Figure 1a, where the two CFBGs are identical (i.e., similar reflection ratio and bandwidth). The grating pitch Λ(z) of the CFBG gradually increases or decreases along the fiber axis (*z* direction), resulting in a gradual change in the reflection wavelength. The phase-matching condition of the CFBG can be deduced from a uniform FBG [18] to: $\lambda (z)=2n_{eff}(z)\Lambda (z)$, where λ(z) and $n_{eff}(z)$ denote the resonant wavelength and the effective index of the fundamental core mode, respectively, and Λ(z) represents the grating pitch of the CFBG. For linear chirped CFBG, the grating pitch varies linearly along the fiber axis according to $\Lambda (z)=\Lambda_{0}+Cz$; where $\Lambda_{0}$ represents the grating pitch at the starting position and C represents the chirp rate. When two identical CFBGs are inscribed in the fiber core one by one, the reflected light from the two CFBGs will result in an interference pattern in the frequency domain according to the white-light interference principles. Assuming the two CFBGs have the same reflection rate of *R*_0_, and the distance between the edges of the two CFBGs is *L*_0_. The reflection spectrum $I_{R}(\lambda )$ of the CFBG-based FPI can be expressed as [19]:

(1) $$I_{R}(\lambda )=I_{0}(\lambda )\frac{R_{0}+R_{0}\eta -2R_{0}\sqrt{\eta }\cos\;[\frac{4\pi n_{eff}L_{0}}{\lambda }+\varphi_{0}]}{1+R_{0}^{2}\eta -2R_{0}\sqrt{\eta }\cos\;[\frac{4\pi n_{eff}L_{0}}{\lambda }+\varphi_{0}]},$$

where $n_{\mathrm{eff}}$ and *η* denote the average effective index and the transmission coefficient of the cavity. For the CFBG-based FPI (intrinsic FPI), the transmission coefficient *η* is ~1 since the optical loss of the cavity is close to zero. $I_{0}(\lambda )$ represents the incident spectrum within the reflection bandwidth of the CFBG and $\varphi_{0}$ denotes the initial phase difference of the two reflected beams. As such, the reflection spectrum can be represented by:

(2) $$I_{R}(\lambda )=I_{0}(\lambda )\frac{2R_{0}-2R_{0}\cos\;[\frac{4\pi n_{eff}L_{0}}{\lambda }+\varphi_{0}]}{1+R_{0}^{2}-2R_{0}\cos\;[\frac{4\pi n_{eff}L_{0}}{\lambda }+\varphi_{0}]},$$

It has been demonstrated that a higher *Q* factor (defined as *Q* = *λ*/Δ*λ*) of the resonant peak/dip renders higher resolution and lower errors [6]. As such, small temperature or strain drift can be precisely determined by tracking the resonant peak/dip within the interference spectrum. Figure 1b shows the simulated reflection spectra of the CFBG-based FPIs with different *R*_0_ of 0.2, 0.4, and 0.8, (assuming the cavity length is 10 mm), where the FWHMs are 0.031 nm, 0.021 nm, and 0.006 nm, respectively. Figure 1c exhibits the reflection spectra of the FPIs with different cavity lengths of 5 mm, 10 mm, and 15 mm (assuming the reflection of CFBG is 0.2), where the corresponding FWHMs are 0.063 nm, 0.031 nm, and 0.021 nm, respectively. As such, for high-resolution temperature/strain sensing, increasing the reflection ratio *R*_0_ and cavity length *L*_0_ are both effective.

The increases in cavity length *L*_0_ reduce the free spectrum range (FSR) accordingly (FSR = *λ*^2^/2*nL*_0_). It is well known that when the drift of the resonant peak/dip is larger than the FSR, the resonant peak/dip can be hardly identified and tracked, resulting in a limited dynamic measurement range. As a result, the conventional spectrum interrogation approach can hardly balance the resolution and dynamic range of the measurement while for the proposed CFBG-based FPIs, the envelope of the interference spectrum may be tracked for large dynamic range measurements since the envelope is essentially the reflection spectrum of the CFBG, which manifests as a single peak with nearly infinite FSR. Thus, we can develop a direct spectrum-domain interrogation method (i.e., the dual-mode spectrum interrogation approach) for both the sharp resonant dip and envelope to identify the tiny external changes within large dynamic ranges.

## 3. Sensor Fabrication

**Figure 2:**
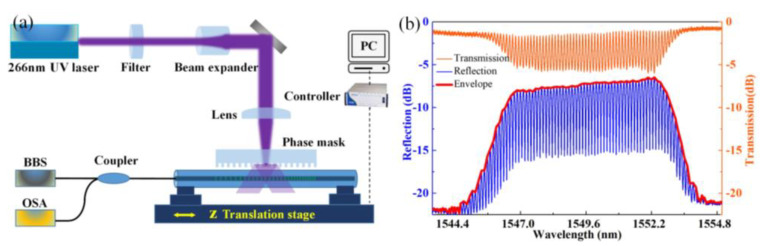
(**a**) Schematic diagram of the CFBG preparation system, including a UV laser operating at 266 nm, a chirped phase mask, and electronic scanning stages; (**b**) Reflection (blue line) and transmission (orange line) spectra of the prepared FPI_1_ sensor with a cavity length *L*_0_ of ~6.9 mm.

A chirped phase mask with a pitch of 1070 nm and a chirp rate of 10 nm/cm is employed for CFBG inscription. A 266 nm, pulse UV laser with pulse width and repetition ratio of 1.5 nm and 10 kHz is employed for inscription. The resulting center wavelength of CFBG is ~1550 nm, according to the grating equation λ(z)=2nΛ(z), and the reflection bandwidth of the CFBG is proportional to scanning length *L*. The scanning setup for CFBG fabrication is sketched in Figure 2a, which is similar to [20]. A CFBG (CFBG_1_) is first inscribed in a hydrogen-load single-mode fiber (SMF-28). The scanning length (i.e., CFBG_1_ length (*L*)) is precisely controlled at ~5.4 mm, resulting in a reflection bandwidth of ~7.8 nm. Then, the fiber is moved axially (*z* direction) for a distance (*L*_0_ = 6.9 mm) and the same inscription process is repeated, with the same CFBG (CFBG_2_) inscribed. In this process, the variation in the grating pitches Λ(z) for the two CFBGs are in the same direction, resulting in a similar cavity length *L*_0_ for each reflection wavelength (as sketched in Figure 1a). Light launched into the fiber will be reflected by the two CFBGs and the reflected light will interfere with the frequency domain (blue line within Figure 2b). Inferring from the transmission spectrum (orange line within Figure 2b), the reflection of the two CFBGs is ~30%. The FSR of the interference spectrum is identified as ~0.12 nm, which agrees well with the designed cavity length (*L*_0_) of ~6.9 mm. The FWHM Δλ of the CFBG-based FPI (FPI_1_) is identified as ~0.036 nm, corresponding to a *Q* factor of ~4.3 × 10^4^. The sensor is finally annealed in a 120 °C oven for 12 h to get a stable spectrum.

## 4. Sensing Performance and Discussion

### 4.1. High-Resolution and Large-Dynamic Range Temperature Sensing

**Figure 3:**
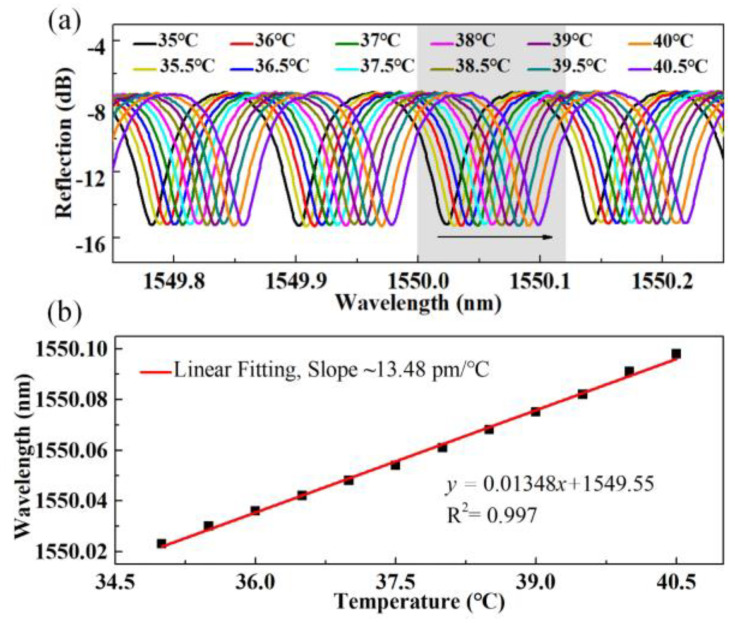
(**a**) The resonant dip evolution of FPI_1_ with temperature increasing from 35 to 40.5 °C in steps of 0.5 °C; (**b**) The wavelength of the tracked resonant dip versus the temperature.

Proof-of-concept temperature tests are then carried out, where a high-precision (0.1 °C) digital temperature oven that is similar to [21] is employed and an ultrahigh-resolution (0.005 nm) optical spectrum analyzer (OSA: Yokogawa, AQ6380, Tokyo, Japan) and a supercontinuum source (YSL-SC-5) are employed for online monitoring of the temperature-induced wavelength drift. FPI_1_ is placed in the center of the oven and the temperature is increased from 35 to 40.5 °C in steps of 0.5 °C. Figure 3a shows the variation in a resonant dip located at ~1550 nm grey-shaded area) versus the temperature. Between each temperature step (0.5 °C), ~0.007 nm wavelength drift can be clearly identified. Figure 3b shows the experimental and linear fit results, where the temperature sensitivity is ~13.48 pm/°C, with *R*^2^ of 0.997. It is worth noting that the proposed sensor is sensitive to both temperature and strain, as such, the setup for temperature tests must be carefully designed to maintain the accuracy and reliability of the tests. We place the sensor in a temperature oven with two sides opened for the sensor pigtails. The pigtails of the sensor are naturally released to reduce the strain effects.

**Figure 4:**
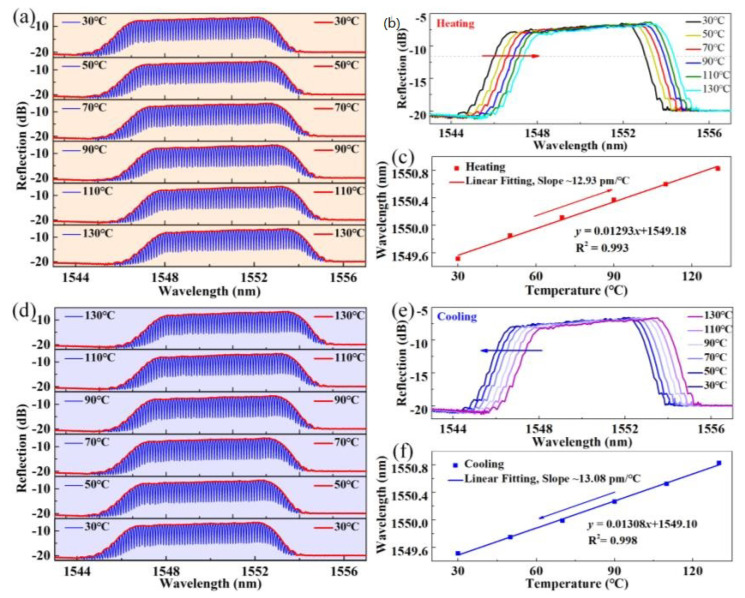
(**a**) The reflection spectrum and (**b**) spectral envelope evolutions of FPI_1_ with temperature increasing from 30 °C to 130 °C; (**c**) The center wavelength of the reflection spectral envelope versus the temperature; (**d**) The reflection spectrum and (**e**) spectral envelope evolutions of FPI_1_ with temperature decreasing from 130 °C to 30 °C; (**f**) The center wavelength of the reflection spectral envelope versus the temperature.

The lengthening of the cavity can improve the spectrum *Q* factor and thus the temperature resolution while the FSR of the spectrum is reduced accordingly, restricting the temperature dynamic range. According to the aforementioned solution, the envelope of the interference spectrum may be tracked for large dynamic range measurements. To verify this approach, temperature tests within a larger dynamic range are then carried out. The temperature is increased from 30 °C to 130 °C in steps of 20 °C, and the reflection spectrum evolution is recorded and plotted in Figure 4a. The envelope of the reflection spectrum can be routinely extracted as shown in Figure 4b. As long as the envelope is extracted, the center wavelength of the envelope peak can be easily extracted. By tracking the center wavelength of the envelope versus temperature change, temperature information can be obtained and calibrated. Figure 4c shows the tracked center wavelength of the envelope versus the temperature, where a sensitivity of 12.93 pm/°C is obtained by a linear fit (*R*^2^ = 0.993). The cooling process is also monitored and the result is shown in Figure 4d–f. A temperature sensitivity of ~13.08 pm/°C (*R*^2^ = 0.998) is obtained. The little deviation in temperature response between the heating and cooling processes may be attributed to thermal stress release as well as experimental errors.

### 4.2. High Resolution and Large Dynamic Range Strain Sensing

**Figure 5:**
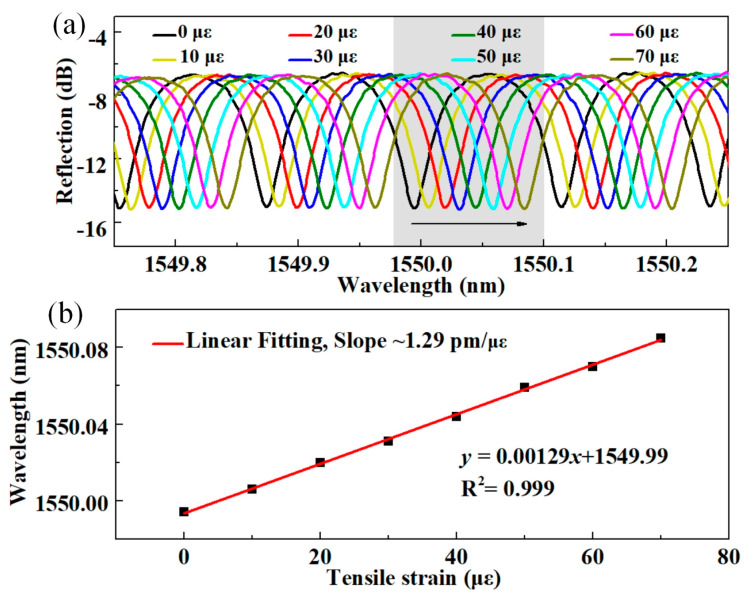
(**a**) The resonant dip evolution of FPI_1_ with tensile strain increasing from 0 με to 70 με; (**b**) The wavelength shifts of the tracked resonant dip versus applied strain.

The proposed sensor and spectrum interrogation approach can also be employed for other measurands, such as strain. The sharp resonant dip within the interference spectrum, as well as the spectrum envelope (i.e., the spectrum of CFBG), will shift with tensile strains due to the elastic-optic effect as well as the lengthening of the cavity. As a proof-of-concept, the strain response of the CFBG-FPI (FPI_1_) is then studied experimentally at room temperature (25 °C), the setup of which is similar to our previous work [12]. To reduce temperature crosstalk, the sensor is placed in a thermostatic oven (temperature stability: 0.1 °C) with two sides opened for the sensor pigtails. The applied strain is increased from 0 µε to 70 µε in steps of 10 µε and the resonant dip at ~1550 nm is marked and tracked (grey-shaded area). At each step, ~0.013 nm wavelength shift of the resonant dip can be clearly identified. Figure 5 shows the tracked wavelength of the dip versus the applied strain, where a sensitivity of ~1.29 pm/µε can be obtained by linear fit (*R*^2^ = 0.999).

It is worth noting that the strain resolution of the sensor (FPI_1_) is higher than 10 µε. While limited by the experimental setups, higher resolution tests need to be further designed and constructed. This work merely gives a proof-of-concept experimental illustration.

**Figure 6:**
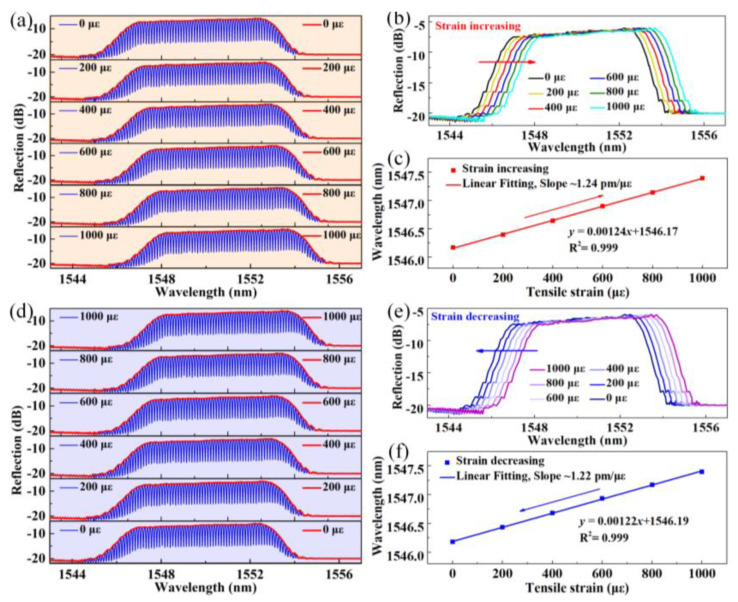
(**a**) The reflection spectrum and (**b**) spectral envelope evolutions of FPI_1_ with strain increasing from 0 με to 1000 με; (**c**) The center wavelength of the reflection spectral envelope shifts versus applied strain; (**d**) The reflection spectrum and (**e**) spectral envelope evolutions of FPI_1_ with strain decreasing from 1000 με to 0 με; (**f**) The center wavelength shifts versus applied strain.

For larger dynamic range strain sensing, the dual-mode spectrum interrogation approach can be employed. Figure 6a,d illustrates the recorded reflection spectrum evolutions with strain increasing (0–1000 µε) and decreasing (1000 µε to 0 µε) in a step of 20 µε. The envelopes of the reflection spectra are extracted and shown in Figure 6b,e. The center wavelength of the envelope varies with the applied strain, where a sensitivity of ~1.24 pm/με is obtained by a linear fit of the data (*R*^2^ = 0.999). The sensitivity is comparable to the results obtained by tracking one of the resonant dips within the interference spectrum.

### 4.3. Performance Improvement

**Figure 7:**
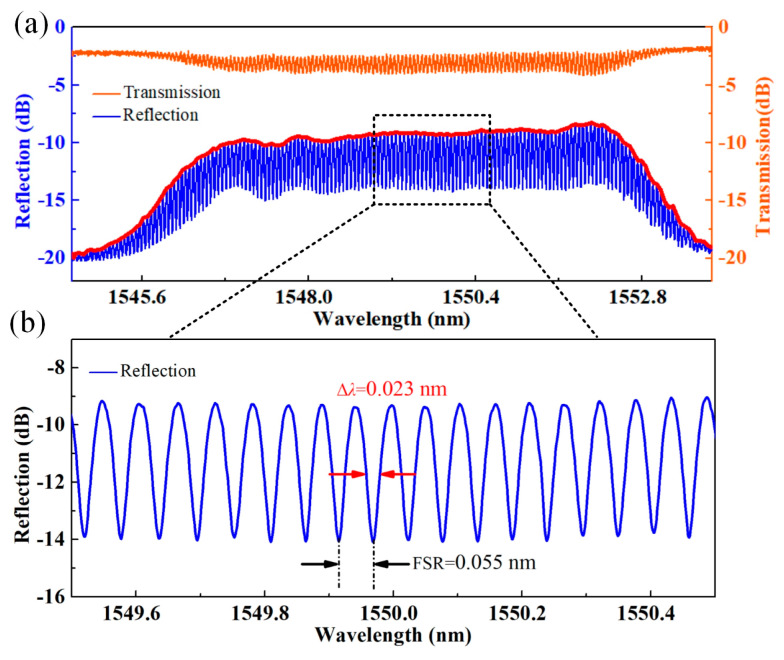
(**a**) Reflection (blue line) and transmission (orange line) spectra of the FPI_2_ with a cavity length of ~15.1 mm, the red lines represent the envelop of the spectrum; (**b**) Enlarged view of the reflection spectrum, where the bandwidth Δλ and FSR are ~0.023 and ~0.055 nm, respectively.

A higher Q-factor CFBG-based FPI (FPI_2_) is then prepared to improve the measurement resolution further. The cavity length (*L*_0_) is increased to ~15.1 mm and the corresponding reflection (blue line) and transmission spectra (orange line) are shown in Figure 7a. Figure 7a is an enlarged view of the reflection spectrum near 1550 nm, where we can see that the FSR and bandwidth (Δλ) of the resonant dip are reduced to ~0.055 nm and ~0.023 nm, respectively. The *Q* factor of the resonant (interference) dip is calculated as ~0.67 × 10^5^. Such a high Q-factor resonant dip can be tracked to distinguish smaller temperature drifts.

**Figure 8:**
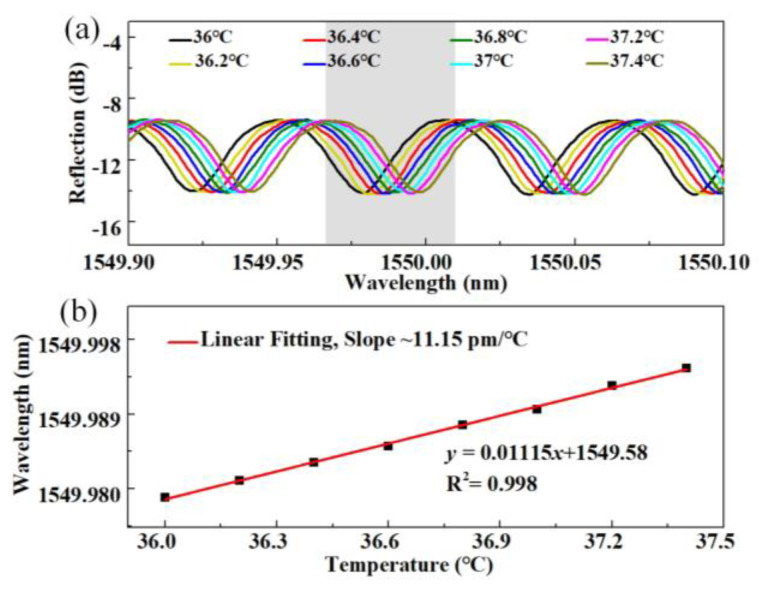
(**a**) The resonant dip evolution of FPI_2_ with temperature increasing from 36 °C to 37.4 °C (temperature of most living bodies); (**b**) The wavelength of the tracked resonant dip versus the temperature.

The FPI_2_ is then employed for smaller temperature drift identifications. The temperature is increased from 36 °C to 37.4 °C (temperature of most living bodies) in a step of 0.2 °C, and the spectrum evolution is shown in Figure 8a. Figure 8b shows the tracked dip near 1550 nm versus the temperature, where a smaller wavelength drift of ~0.002 nm can be identified during each temperature step. We can clearly see that the resonant dip within the reflection spectrum shifts toward longer wavelengths with the temperature increasing, and the temperature sensitivity is ~11.15 pm/°C by a linear fit of the experimental results (*R*^2^ = 0.998). This result indicates that the proposed sensor can precisely identify a temperature change of less than 0.2 °C. Further on, we experimentally studied the long-term stability of the sensor by placing the sensor in a constant environment (40 °C) for three hours. The wavelength of the tracked dip was recorded, where the average wavelength and standard deviation were calculated to be 1550.157 nm and 0.00135 nm, respectively. The results indicate the excellent long-term stability of the sensor.

It is worth noting that: (1) the temperature resolution of FPI_2_ is higher than 0.2 °C, the presented experiment is limited by the accuracy and resolution of the employed temperature oven; (2) The cavity length *L*_0_ of the FPI can be routinely increased further by controlling the translation distance of the fiber relative to the phase mask. Since the in-fiber cavity has extremely low loss (*η* ≈ 1), the cavity can be lengthened infinitely as long as it provides suitable spectrum readout setups; (3) The reflection of the CFBGs can also be further increased by repeat scanning or increasing the power of the UV lasers. The two strategies (i.e., (2) and (3)) together with the *Q* factor of the resonant (interference) dip can be significantly improved, yielding an ultrahigh resolution and a large dynamic range sensor; (4) Temperature and strain crosstalk existed in the proposed sensor structure. However, the crosstalk can be resolved using a lot of strategies, including compensation and coefficient matrix strategies [22,23,24,25].

## 5. Conclusions

In conclusion, we present a dual-mode direct spectrum interrogation approach for simultaneously sensing high resolution and large dynamic range temperature/strain. As a proof-of-concept demonstration, temperature and strain resolution of 0.2 °C and 10 µε within dynamic ranges of 30–130 °C and 0–1000 µε, respectively, are experimentally illustrated. The resolution can be improved further by increasing the reflectivity of the CFBG and the cavity length. The proposed sensing schema can resolve the conflict between the resolution and the dynamic range of fiber-optic sensors effectively and can be extended to other sensors and measurands. However, the reflection of the CFBG is not high enough in this work, hence restricting further improvement of the *Q* factor and thus the resolution. Future efforts can be made to optimize the CFBG inscription process to improve the reflection of the CFBGs. An automatic dual-mode spectrum interrogation algorithm is also urgently needed for practical applications.

## Data Availability

Data are available from the corresponding author and can be provided upon appropriate request.
